# On the Spontaneous Expulsion and Artificial Extraction of the Placenta before the Child, in Placental Presentations

**Published:** 1845-05

**Authors:** 


*On the Spontaneous Expulsion and Artificial Extraction of the Placenta before the Child, in Placental Presentations.*—Professor Simpson read to the Medico-Chirurgical Society of Edinburgh, Dec. 4th, 1844, a paper on the expulsion and extraction of the placenta before the child, in cases of unavoidable hemorrhage. He showed, that in common cases of presentation of the placenta, when managed according to the rules generally followed under the circumstances, the mortality among mothers was very great. Out of 174 cases, tabulated from different authors, by Dr. Churchill, this complication had proved fatal to 48 mothers; and a more extensive table of 339, drawn up by Dr. S. himself, presented a mortality of 115 mothers:—or, one out of every three died.

In contrast with these statistics, Dr. S. brought forward a number of cases, (some previously recorded, and others collected from private sources,) in which the placenta had come away before the infant, either expelled by the natural efforts alone, or in consequence, in several instances, of the reputed bad management of the accoucheur. The number of cases collected was 120 in all. Out of these only eight mothers died, or one in fifteen. In two of these, the cause of death was not stated by the reporter; in three, the patient perished from puerperal fever; and two only were alleged to have died from hemorrhage. In one of these two last cases, the hemorrhage ceased as soon as the placenta was separated, but too late to save the woman.

The same cases also show that, though much blood may have been escaping *before* the placenta comes away, yet as soon as the separation is *complete*, the hemorrhage usually ceases, or becomes very trifling. A complete separation of the placenta is thus proved to be far less dangerous than a partial one,—a fact that at first may appear somewhat paradoxical, but which is readily explained by the construction of the fœtal placenta. The hemorrhage comes chiefly from the placenta itself. When it is only partially separated from the uterus, the blood enters freely by the adherent portions, and escapes as freely from the surface of the portion of placenta that is detached.

From a consideration of these facts, Dr. S. was led, four years ago, to propose to the obstetrical society, whether in cases of hemorrhage from placental presentations, we should not sometimes adopt the practice of extracting the placenta, in order to arrest unavoidable hemorrhage, leaving the fœtus to be expelled by the natural efforts of the uterus, or otherwise. Dr. S. stated he had adopted this procedure, in one case, with perfect success, the placenta having been extracted two hours before the birth of the child. This method, he thought, would be found particularly applicable to those sets of cases in which turning or rupture of the membrane is inexpedient or impracticable; as, in cases where hemorrhage occurs to an alarming extent, while the os uteri is still small and rigid; in unavoidable hemorrhage in first labors; in placental presentations, when the patient's strength is already so sunk, from the flooding, as not to allow, without danger, of immediate turning or forcing delivery; in cases where the child is known to be dead, &c., &c.

At the subsequent meeting of the Society, (Jan. 8, 1845,) Dr. S. asserted his claims to have originated the plan of treatment described above, and stated that it had been discussed yearly ever since he was elected to the Chair of Midwifery in the University,—was well known to most of his obstetric brethren in Edinburgh,—and had been, in 1841, formally brought by him before the Obstetric Society. He was sorry to be obliged to state these points in his own behalf on such a subject, but he had been advised that silence upon the matter would be highly unjust, both as regarded himself, and as regarded the Society.—*Lond. & Edin. Month. Jour. Med. Sci.*

